# *csrnp1a* Is Necessary for the Development of Primitive Hematopoiesis Progenitors in Zebrafish

**DOI:** 10.1371/journal.pone.0053858

**Published:** 2013-01-09

**Authors:** Jaime Espina, Carmen G. Feijóo, Camila Solís, Alvaro Glavic

**Affiliations:** 1 Departamento de Biología, Facultad de Ciencias, Universidad de Chile, Santiago, Chile; 2 Departamento de Ciencia Biologicas, Facultad de Ciencias Biologicas, Universidad Andres Bello, Santiago, Chile; University of Colorado, United States of America

## Abstract

The CSRNP (cystein-serine-rich nuclear protein) transcription factors are conserved from *Drosophila* to human. Functional studies in mice, through knockout for each of their paralogs, have resulted insufficient to elucidate the function of this family of proteins in vertebrate development. Previously, we described the function of the zebrafish ortholog, Csnrp1/Axud1, showing its essential role in the survival and proliferation of cephalic progenitors. To extend our understanding of this family, we have studied the function of its paralog *csrnp1a*. Our results show that *csrnp1a* is expressed from 0 hpf, until larval stages, particularly in cephalic territories and in the intermediate cell mass (ICM). Using morpholinos in wild type and transgenic lines we observed that Csrnp1a knockdown generates a mild reduction in head size and a depletion of blood cells in circulation. This was combined with *in situ* hybridizations to analyze the expression of different mesodermal and primitive hematopoiesis markers. Morphant embryos have impaired blood formation without disruption of mesoderm specification, angiogenesis or heart development. The reduction of circulating blood cells occurs at the hematopoietic progenitor level, affecting both the erythroid and myeloid lineages. In addition, cell proliferation was also altered in hematopoietic anterior sites, specifically in *spi1* expression domain. These and previous observations suggest an important role of Csnrps transcription factors in progenitor biology, both in the neural and hematopoietic linages**.**

## Introduction

All vertebrates, including teleosts, have two waves of hematopoiesis occurring sequentially during development. The first is the so-called primitive hematopoiesis, which produces mainly erythrocytes and primitive macrophages. The second is the definitive hematopoiesis, which generates long-term hematopoietic stem cells (HSC) capable of unlimited self-renewal and which is able to generate all mature hematopoietic lineages. In zebrafish, primitive hematopoietic cells arise from two distinct territories of the lateral plate mesoderm (LPM), anterior and posterior, which can be evidenced by the expression of the early hematopoietic marker *tal1/scl* (T-cell acute lymphocytic leukemia 1) [1]. The anterior LPM gives rise to the rostral blood island (RBI), while hematopoietic cells from the posterior LPM migrate ventrally towards the midline to fuse, forming the intermediate cell mass (ICM) [2]. The RBI generates mainly macrophages and endothelial cells whereas cells from the ICM differentiate as endothelial cells of the trunk vasculature, neutrophils, and proerythroblasts. The primitive erythroblast population arises from a subset of posterior *tal1* expressing cells that also express the Krüppel-like transcription factor *klf4* [3] and the zinc finger transcription factor *gata1* [4]. These proteins are expressed bilaterally in the posterior LPM and promote the expression of genes essential for erythroid differentiation like *hbae1* [5]. On the other hand, from a subset of anterior *tal1/scl* expressing cells, which also express *spi1*, mature macrophages and neutrophils arise, which are recognized by the expression of markers like lymphocyte cytosolic plastin1 *lcp1*, and myeloid-specific peroxidase *mpx* respectively [6].

The CSRNP family of transcription factors has been conserved from *Drosophila* to humans. These proteins do not have any recognized domain or structural motif recorded previously in any database. However, *in silico* analysis has identified a motif of three regions at the amino terminus clearly present in every member of this family. The first region is rich in serine followed by a basic domain and a final cysteine-rich region [7]. The subcellular localization of *csrnp* gene products, in the three mouse paralogs, CSRNP-1, 2 and 3, as well as in the single *Drosophila* ortholog described, DAxud1, is the nucleus [7], [8]. This, together with the fact that the mouse protein can activate transcription in a Gal4 fusion assay [7] suggests that these proteins have transcription factor characteristics and might behave as such. The *in vivo* function of these proteins has been studied by obtaining single knockout mice for each of the three paralogs and successively through combinations of double and triple knockout animals. The mutant mice were indistinguishable from wild type in every aspect analyzed with the exception of the triple KO mice, which died neonatally [7]. In *Drosophila*, there is only one member of the CSRNP family, DAxud1. Functional studies revealed that DAxud1 regulates distinct aspects of the cell cycle and apoptosis, suggesting that it could behave as a tumor suppressor [8]. Its overexpression causes, through changes in CDK1 activity, a G2/M cell-cycle arrest, and apoptosis induction through the activation of the JNK pathway; in addition DAxud1 knockdown increases the proliferation of imaginal disc cells [8]. This last result is in correspondence to previous publications showing that human AXUD1 message decrease in cancers associated with mutations in the Wnt pathway transducer AXIN1 [9]. Moreover, it has been found that DAxud1 interacts genetically with *Spt5* [10], [Glavic unpublished results]. The *spt5* product together with Spt6, are part of the transcription elongation factor DSIF. In zebrafish this elongation complex, and in particular *Spt5*/Foggy, regulate erythropoiesis through *gata1* expression [11].

Two sequences homologous to DAxud1 have been identified in zebrafish, with 69% and 63% of amino acidic identity [12]. Previously, we reported the functional analysis of the closest sequence to the *Drosophila* DAxud1 present in zebrafish, the Csrnp1/Axud1 gene, showing that it is essential for the expansion and survival of cephalic neural progenitors [12]. Here, we present the expression and functional analysis of the second zebrafish paralog, *csrnp1a*. Our expression studies showed that *csrnp1a* is present from cleavage stages and, later on, its transcription becomes dynamically restricted to two territories, the cephalic domain and the caudal region, specifically at the ICM. Knockdown analysis indicated that *csrnp1a* morphants display a mild head size reductions and a remarkable decrease in circulating blood cells. This phenotype is probably the result of a decrease in the erythroid transcription factor *gata1*, a change of the distribution in the *spi1* signal at 12 somites and a diminished cell proliferation in *spi1* territories, all of which are indicative of a role for *csrnp1a* in the development of primitive blood lineages. Together, these results expand our understanding about the role of the CSRNP protein family in vertebrate development.

## Results

### *csrnp1a* Expression Pattern

In previous work, our laboratory identified the zebrafish *csrnp1* gene and characterized its function [12]. During the analysis of related sequences, a second member of the Csnrp family, Csnrp1a, was also identified (accession number: XM_683666.4). This protein of 514 amino acids has 63% identity at amino acid level to the *Drosophila* AXUD1 and its coding sequence maps to chromosome 2 of zebrafish at locus NC_007113. The gene spans 14.2 kb, and consists of 5 exons that generate a predicted messenger of 2941 bp (Fig. S1).

**Figure 1:**
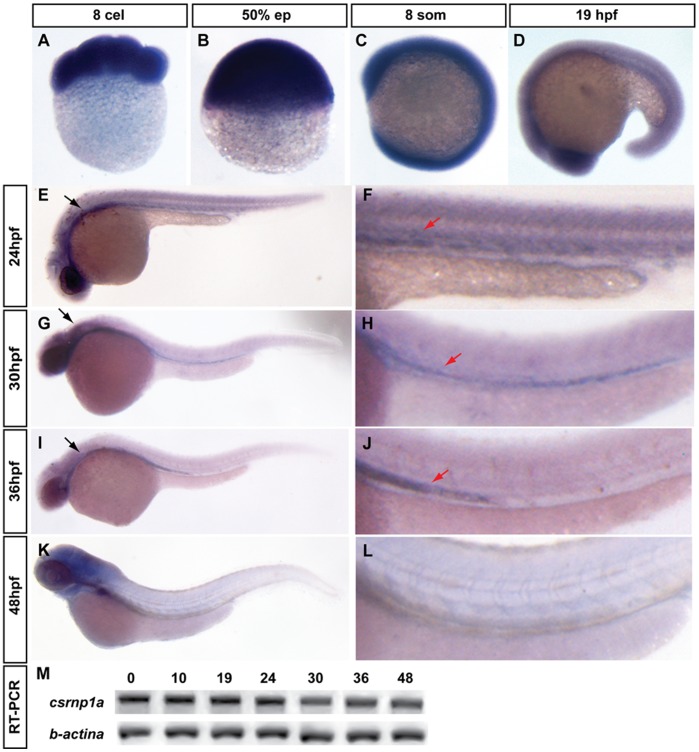
Expression pattern of *csrnp1a* in zebrafish embryonic development. Early embryos show a ubiquitous signal of *csrnp1a* (A to D). Latter Stages (E to L) show an extensive anterior expression in the head region (black arrow) and a caudal, much localized expression, at the intermediate cell mass (ICM) (red arrow) (all are lateral views; A to C animal pole to the top; D to L anterior to the left). (F, H, J, L) Magnifications showing the expression of *csrnp1a* at ICM. Note the dynamic extension and retraction of its expression. (M) *csrnp1a* RT-PCR; the numbers above the gel correspond to developmental stages (as hpf).

To identify the temporal expression pattern of *csrnp1a* we performed RT-PCR using cDNA templates obtained from embryos at 0, 10, 18, 24, 30, 36 and 48 hours post fertilization (hpf) (Fig. 1M). This showed that *csrnp1a* is maternally inherited and expressed through all the time points analyzed. To obtain its spatial expression pattern, we performed *in situ* hybridizations at different stages. At early stages, *csrnp1a* is distributed over the entire embryo (Fig. 1A, B, C). Later on, at 19 hpf, its expression begins to be restricted to ventral regions along the A-P axis (Fig. 1D). Between 24 and 36 hpf, it is possible to detect its ventral expression in anterior cephalic tissues and in posterior regions (Fig. 1E to L, black and red arrows respectively). In the anterior region, the expression is initially ubiquitous (Fig. 1C, D) and then limited to the ventral domain (Fig. 1E, G, I, black arrows). The posterior expression is progressively circumscribed to the intermediate cell mass (ICM), the primary hematopoietic tissue in zebrafish. In this territory, the expression of *csnrp1a* is very dynamic. At 24 hpf, an initial extension is detected in the ICM (Fig. 1E, F), which reaches its maximum at 30 hpf (Fig. 1G, H). Later, at 36 hpf, the distribution of mRNA becomes gradually restricted (Fig. 1I, J), disappearing at around 40 hpf (Fig. 1K, L). The comparison between *csrnp1* and *csrnp1a* reveals that although both genes are expressed in cephalic domains, paralog specific expression territories can be assigned. Particularly we detected *csrnp1a* transcription in the ICM at the time when hematopoietic progenitors expand and the initial blood cells reach circulation. This observation, together with the fact that the ICM is a primary site of proliferation and differentiation of hematopoietic cells [13], has lead us to postulate a role for *csrnp1a* in hematopoiesis.

### Knockdown of *csrnp1a* in Developing Embryos

**Figure 2:**
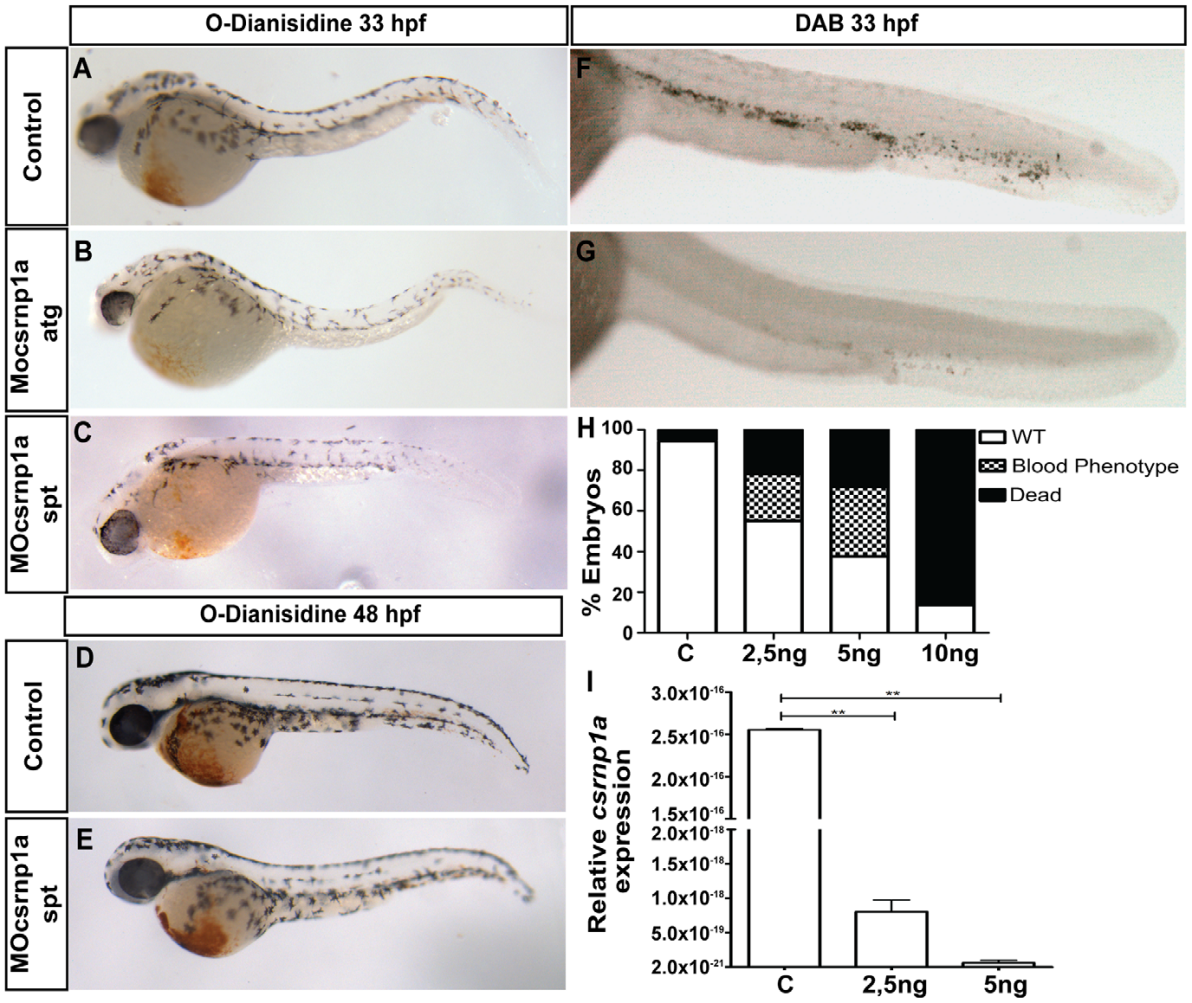
*csrnp1a* knockdown decreases the number of blood cells in circulation. O-Dianisidine stain labels erythrocytes (A, B, C, D, E) and Diaminobenzidine (DAB) blood cells in general at the ICM (F and G). The injection of *csrnp1a* morpholino produces a dramatic reduction of blood cells in 34% of the injected embryos (n = 16/47) at 33 hpf (A, B, C, F, G) which is reverted at 48 hpf (D, E) (all are lateral views, anterior to the left). (H) Chart summarizing the morpholino dose-phenotype quantifications of 26 hpf control embryos (n = 71) and injected with 2,5 ng (n = 69), 5 ng (n = 85) or 10 ng (n = 87) of Mocsrnp1a spt morpholino. (I) qPCR with primers located in exon 2 and 3 of 24 hpf control embryos (C) and splicing morphants injected with two concentrations, 2,5 and 5 ng per embryo, showing the relative expression of *csrnp1a* message, which decreases significantly in a dose-dependent manner (t student p<0,01).

To test the aforementioned hypothesis, we injected morpholino oligonucleotides (MOs) designed to suppress the expression of csrnp1a. To avoid or reduce the nonspecific effects of MO injection we examined two different types of morpholinos, one designed to bind to the ATG site (Mocsrnp1a atg), thus inhibiting translation, and the other designed to block the correct splicing of the primary message (Mocsrnp1a spt) through its binding to the donor and acceptor sites at the second and third exons (Fig. S1). To confirm the efficiency of this last morpholino and to test if the morpholino is acting in a dose-dependent manner, we injected the splicing morpholino at 2,5 and 5 ng per embryo, showing by qPCR (Fig. 2I) and RT-PCR (Fig. S2) the dose-dependence reduction at expression at 24 hpf. In addition we checked the decrease of the *csrnp1a* RT-PCR signal in embryos at 30 hpf, observing that *csrnp1a* processed messenger reaccumulate (Fig. S2). Thus this splicing morfolino was no longer effective at this stage. The phenotype resultant of morpholino activity and therefore informative about the role of *csrnp1a*, was a mild head reduction and the notable lack of circulating cells in embryos at 26 hpf. The concentration of morpholino used to achieve these phenotypes was 2.5 and 5 ng per embryo for the ATG and splicing morpholino respectively. This phenotype was generated by both morpholinos in 34% of the injected embryos (Fig. 2A, B, C and quantified in H), suggesting that both morpholinos specifically knocked down *csrnp1a*. The percentage of embryos with the blood phenotype was related with the amount of splicing morpholino injected, being the 5 ng the maximum tolerated before massive embryo death (Fig. 2H). Although we have tried in several opportunities and with different strategies to clone the full length *csrnp1a,* we have not being able succeeded, so the ideal rescue experiment to confirm this assumption is impossible to be carried out at this moment.

On the other hand, to better visualize the reduction of blood cells observed in morphant embryos we used two different stains: O-dianisidine, which reacts with the hemoglobin present in erythrocytes generating an orange precipitate [14] and Diaminobenzidine (DAB), which detects the myeloperoxidase activity in myeloid cells [15], although using longer development of this stain also labels the intermediate cells mass. A significant decrease in both stains was obtained in morphant embryos at 33 hpf, corroborating the *in vivo* observation of reduced blood cells in circulation (Fig. 2A, B, C, F and G). Conversely, and in agreement with the observed reduction of morpholino efficiency at 30 hpf (Fig. S2), we detected a reversion of the blood phenotype in 77,3% of the morphant embryos at 48 hpf (Fig. 2E). Taken together, these results suggest that both myeloid and erythroid lineages are diminished in the *csrnp1a* knockdown condition.

### Csrnp1a Reduction does not affect *csrnp1* Expression

**Figure 3:**
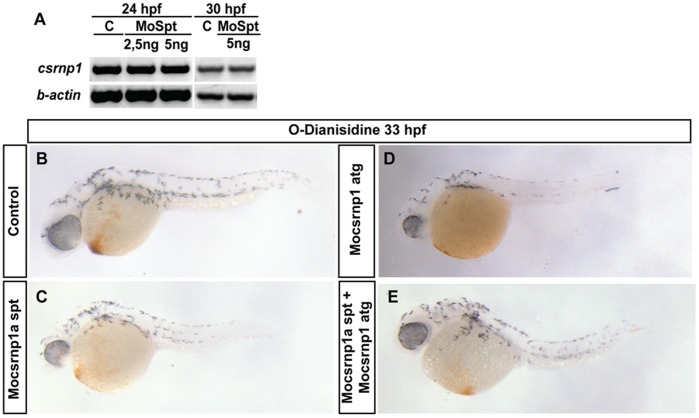
Csrnp1a reduction does not affect *csrnp1* expression, but has redundant function in the appearance of circulating blood cells. RT-PCR against *csrnp1* message in control and *csrnp1a* morphant embryos (A) showing no differences in the *csrnp1* expression at 24 and 30 hpf at the Mocsrnp1a spt concentration used. (B to E) O-dianisidine stain showing the decrease in blood cells in 38,7% of *csrnp1a* injected embryos (C); 22,6% of the *csrnp1* morphant embryos (D), and 64,4% when *csrnp1* and *csrnp1a* morpholino are co-injected (E).

Due to both members of the zebrafish Csnrp family share expression territories, we decided to test if there is any regulation between Csrnp1a and *csnrp1*. To perform this analysis we injected the *csrnp1a* splicing morpholino and analyzed *csrnp1* expression by *in situ* hybridization at two stages, 8 somites and 30 hpf. We did not find any difference in *csrnp1* expression between control and *csrnp1a* morphants (data not shown). This observation was corroborated by RT-PCR of *csrnp1* in *csrnp1a* morphant embryos (Fig. 3A). Likewise, we hypothesized that matching expression territories of Csnrp1 and Csnrp1a at early stages of development could reflect similar functions. To test this we co-injected the *csrnp1a* splicing morpholino with the *csrnp1* atg morpholino [12]. As can be observed in Figure 3, *csrnp1a* knockdown generated the blood cell decrease previously described in 38,7% of the injected embryos (Fig. 3C). Similarly, the *csrnp1* morpholino also generated the reduction in blood cells but only in 22,6% of the injected embryos (Fig. 3D). Importantly, the amount of embryos with the blood phenotype was enhanced up to 64,4% when both morpholino were co-injected (Fig. 3E). This result suggests that both genes may have redundant or complementary functions in the hematopoietic process.

### *csrnp1a* does Not Participate in Early Mesoderm Patterning

**Figure 4:**
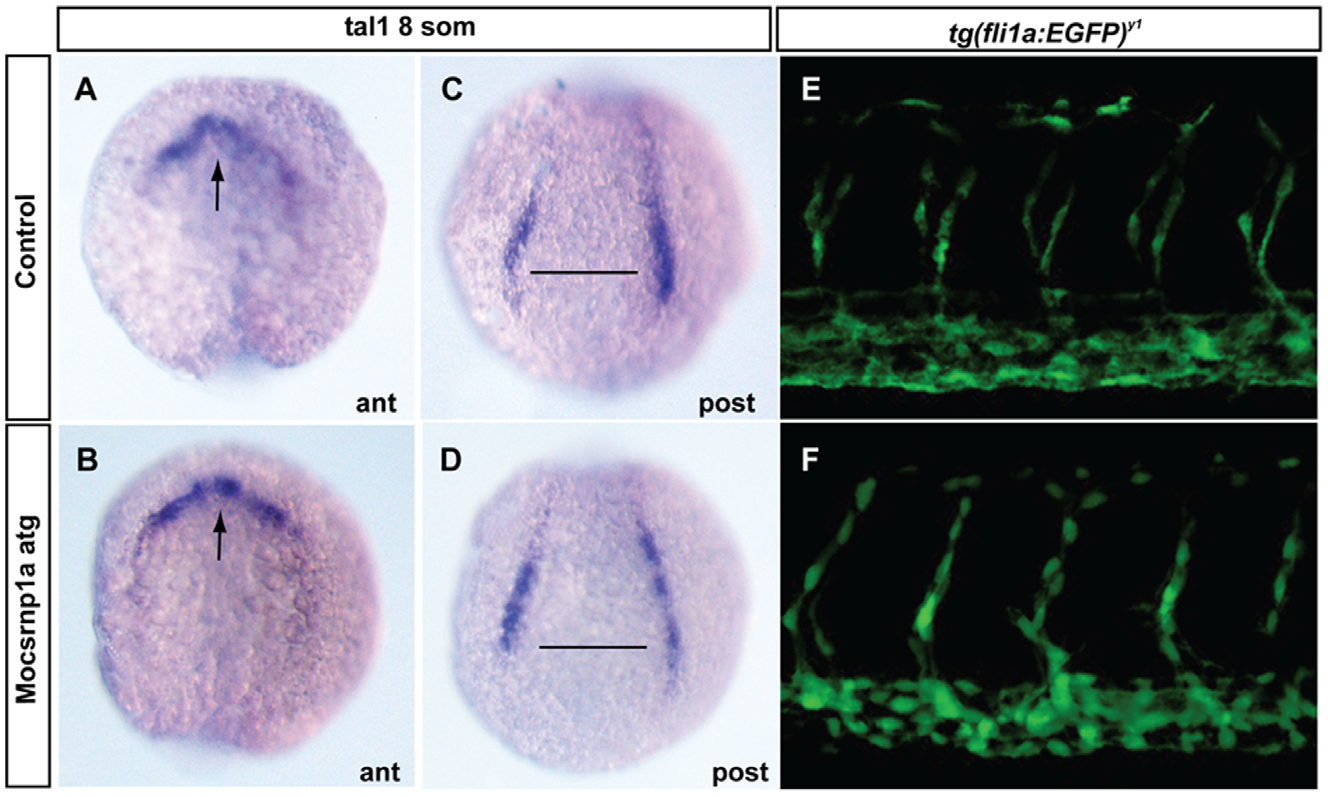
Csrnp1a reduction does not affect the hemato-angiogenic mesoderm. *In situ* hybridization indicating the expression of the hemangioblast marker, *tal1/scl* at 8 somites (A to D) (A, B anterior view; C, D posterior view). Control and morphant 32 hpf transgenic zebrafish embryos expressing GFP under the promoter of the endothelial marker *fli1a* (*Tg(fli1a:EGFP)^y1^*)(E, F) (lateral view, anterior to the left). We did not observe difference in either marker in 100% of the injected embryos (n = 53).

To gain further insight about this phenotype and the specific role of *csrnp1a* on its generation, we decided to examine the earliest stage of hematopoiesis affected in *csnrp1a* morphants. Since hematopoietic cells arise from a distinct mesodermal domain, we first analyzed whether the early specification or patterning of this territory was affected. The expression of the specific mesodermal markers *goosecoid*, *notail* and *gata5* indicated that there were no differences between morphant and control embryos (Fig. S2), excluding the possibility that alterations in mesoderm specification or patterning were responsible for the phenotype described. Next, we examined whether the selection of early progenitors was disturbed in morphant embryos. To this end we analyzed the expression of the earliest hematopoietic marker in zebrafish, T-cell acute lymphocytic leukemia 1, *tal1*, which is expressed in the primitive hematopoietic stem cells and vascular precursors [16]. At 8 somites (13 hpf), approximately 3 hours after progenitor selection, no substantial differences in the expression of this gene was seen between control and morphant embryos (Fig. 4A to D), suggesting that early progenitors of hematopoietic cells were correctly specified. In accordance to this, we observed that blood vessels marked with GFP under the promoter of the endothelial marker *fli1a*, in the transgenic line *Tg(fli1a:EGFP)^y1^*, which develops from subsets of *tal1* positive cells, were correctly formed (Fig. 4E and F). This last result also excludes the possibility that defects in blood circulation arise as a consequence of vessel malformations. Likewise, another factor that could explain the decrease in circulating cells is an extremely reduced blood flow [17]. This possibility was excluded by two indirect approaches. Nitric oxide (NO) is a well-established direct regulator of vascular tone and reactivity, thereby influencing blood flow [17], so we incubated morphant embryos in the NO donor S-nitroso-n-acetyl-penicilamine (SNAP), and tested whether this compound was capable to rescue the morphant phenotype enhancing blood flow. *csrnp1a* knockdown phenotype was not rescue by SNAP treatment (Fig. S3). Second, *in situ* hybridizations using auricular (*amhc*) and ventricular (*vmhc*) probes were used to check for heart malformations that could influence blood flow. Signal of both *amhc* and *vmhc* in morphant and controls embryos were indistinguishable between each other (Fig. S3). Additionally, the number of heartbeats was determined in order to discard heart failure as a source of reduced blood circulation. We detected a marginal, whilst significant, heartbeat reduction in morphant embryos (Fig. S3), but we believe this reduction is too small to generate the observed phenotype. This last result supports the notion that blood circulation defects do not arise as a consequence of heart malformation. Together, these results suggest that a decrease in blood flow is not the cause of the *csrnp1a* morphant phenotype.

### *csrnp1a* is Essential during Erythropoietic Lineage Development

In order to establish if *csnrp1a* function is necessary in the erythropoietic branch of hematopoiesis, we evaluated the expression of *klf4* and *gata1* by *in situ* hybridization. These transcription factors are expressed in the posterior lateral plate mesoderm acting downstream *tal1* in the specification of the erythroid lineage. Also, *klf4* directly activates *gata1* and thus both genes are expressed in the posterior lateral plate mesoderm [3].

**Figure 5:**
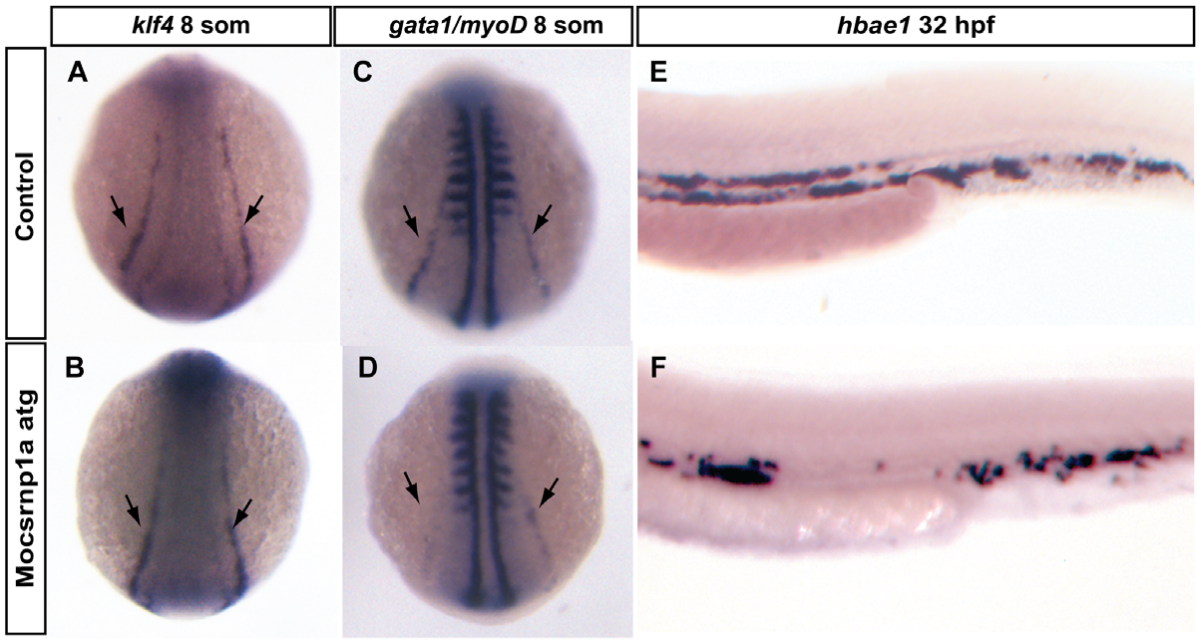
Erythroid/Gata1 precursors are affected by *csrnp1a* knockdown. (A, C, E) Control and (B, D, F) morphant embryos. At 8 somite stage the expression of the erythroid marker *klf4* was not affected in morphant embryos (A, B) (100%, n = 48). However a clear decrease in *gata1* expression was detected in *csrnp1a* deficient embryos (compare C with D) (54,8%; n = 23/42) without major changes in the number of *myoD* stripes (7,1%; n = 3/42, more than 2 stripes), which served as readout of developmental progression. Control embryos showed normal *gata1* expression in 100% of the cases (n = 0/27) and 3,7% have altered *myoD* expression (n = 1/27). In agreement with *gata1* inhibition, at 32 hpf terminal differentiation marker, *hbae1*, was reduced in morphant embryos (E, F) (32,6%; n = 14/43).

At 8 somites, no significant differences in the expression of *klf4* were detected between control and morphant embryos (Fig. 5A, B), suggesting that erythroid precursors were correctly specified at this stage in the differentiation process. In contrast, morphant embryos showed a strong reduction of *gata1* expression (Fig. 5C, D), indicating that *csrnp1a* regulates *gata1* expression and erythropoietic development downstream *klf4*. This inhibition was not the result of a general developmental delay as revealed by the invariable pattern of *myoD* stripes between morphant and control embryos (Fig. 5C, D). Accordingly, we observed that *gata1* inhibition was accompanied by a decline in the amount of mature erythrocytes, which was verified by the diminished expression of embryonic alpha hemoglobin 1, *hbae1* (Fig. 5E, F).

Considering the described role of Csrnp1 in cephalic survival we decided to investigate if the restriction in *gata1* expression and the decrease in mature erythrocytes were due to cell death induction or perturbations in proliferation. Robu et al. (2007) reported that morpholino injections increase cell death activating the p53 pathway [18]. Consequently, we decided to co-inject p53 with *csrnp1a* morpholinos and compare the number of cells labeled with the cell death marker, acridine orange. At 8 somites, morphant embryos displayed a generalized increase in cell death that dropped to control levels when co-injected with p53 morpholino. Importantly, the suppression of cell death produced by p53 and *csrnp1a* morpholino co-injection did not modify the reduction of blood cell in circulation or the phenotype generated by Csrnp1a knockdown (Fig. S4). At this same stage we also checked proliferation by immunohistochemistry against H3-P but no significant change was detected in the *tal1* expressing area (data not shown).

### Csrnp1a is Necessary for Myeloid Lineage Formation

**Figure 6:**
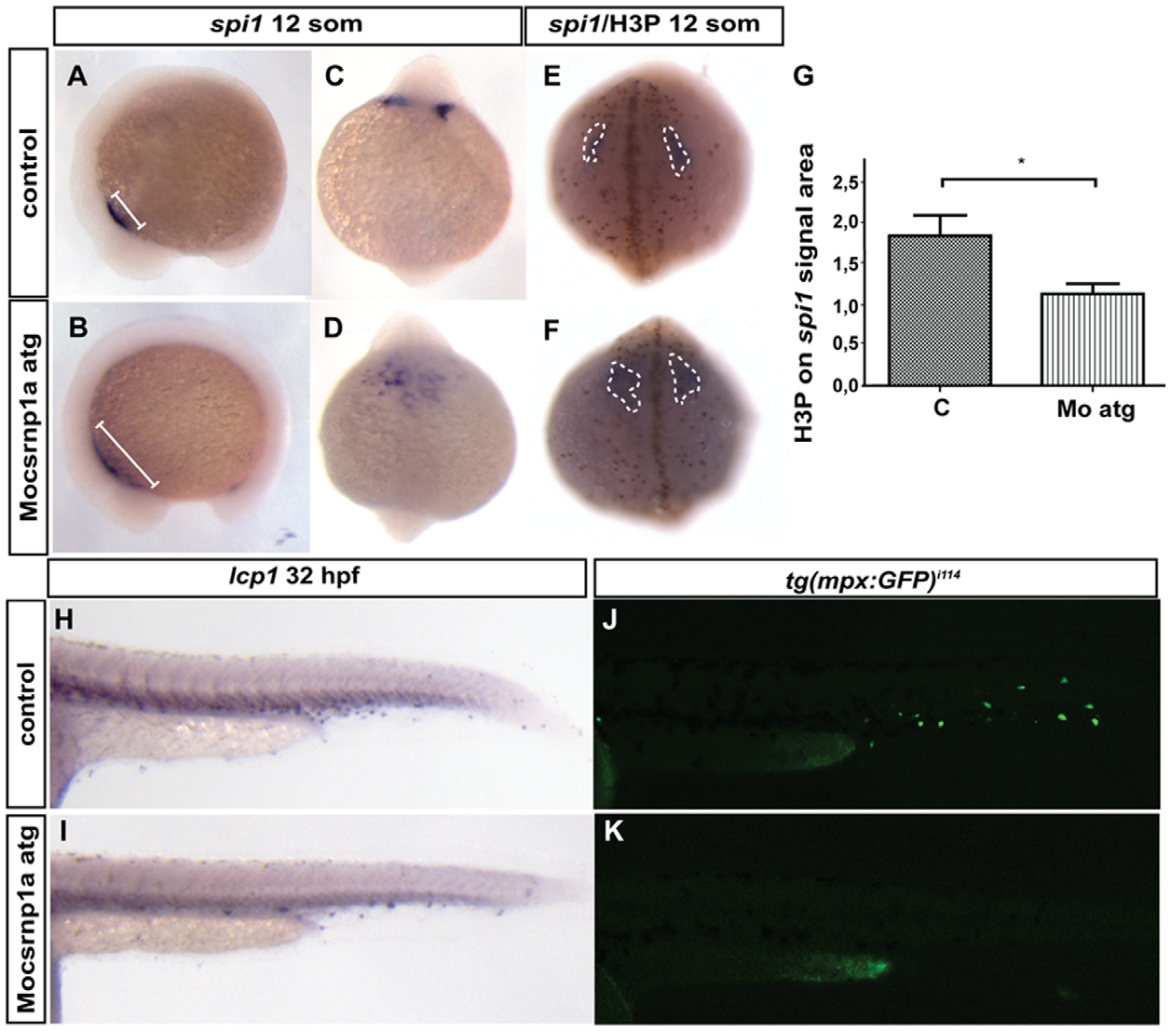
Myeloid lineage is affected by csrnp1a knockdown. Morphants embryos show an alteration in the *spi1* signal (A to D; A, B lateral views anterior lo left; and D, E anterior views). 85,7% of control embryos exhibit normal *spi1* expression pattern (n = 42/49) (A, C), while *csrnp1a* morphants exhibit altered *spi1* expression in 59,2% (n = 61/103) (B, D). The *spi1* changes are accompanied by a significant decrease in proliferation (showed as the number of H3P within the *spi1* signal area) (E, F, and G). Finally, a decrease in the macrophage specific marker *lcp1* (43,8%; n = 14/32) is evidenced by ISH (H, I), and a clear reduction in the neutrophil specific marker *mpo* is evident in the transgenic line expressing GFP under the *mpo* promoter *Tg(Bacmpo:GFP)^i114^* (J, K)(47,2%; n = 25/53). All are lateral views, anterior to the left.

Finally, in attention to the reduction in DAB stain observed in the ICM in morphant embryos, we tested whether *csnrp1a* could also participate in the differentiation of the myeloid lineage. The transcription factor *spi1* was reported to be an early key factor in the process of primitive mielopoyesis in zebrafish [6]. *In situ* hybridization assay showed that *csrnp1a* knockdown modifies *spi1* expression pattern, not by decreasing *spi1* signal but changing its spatial distribution (Fig. 6A to D). At 12 somites, it is possible to observe a scattering of the *spi1* expression domain in *csrnp1a* knockdown embryos; while in control embryos this signal is compact. On the other hand, when cell proliferation was examined, a significant decrease in the number of Phosphorylated-Histone H3 positive cells in the *spi1* expression domain was detected (Fig. 6E to G). These results imply that Csrnp1a knockdown affects also the myeloid branch of primitive hematopoiesis. The effect on early myeloid progenitors was verified by the observation that the number of differentiated myeloid cells, neutrophils and macrophages, drastically decrease in morphant embryos marked with the *lcp1* probe and in transgenic morphant embryos expressing GFP under the *myeloperoxidase* promoter (*Tg(Bacmpx:GFP)^i114^*) (Fig. 6H to K).

## Discussion

### *csrnp1a* Expression during Early Zebrafish Development

The expression pattern of *csrnp1a* is highly dynamic, showing an initially ubiquitous distribution, and then localizing over the ventral side of the embryo, expanding and retracting over the ICM. When we compared this expression with its paralog [12] we can see that *csrnp1a* and *csrnp1* are not identically expressed. However both genes share expression in anterior regions of the neural tube, suggesting that common regulatory mechanisms, perhaps Shh signaling as in the case for *csrnp1*, operate in this territory to control *csrnp1a*. Additionally, *csrnp1a* is transcribed in the intermediate cell mass, which is the site of proliferation and differentiation of primitive hematopoietic cells [13]. Zebrafish has two waves of hematopoiesis, the primitive and definitive. However considering that reductions of blood cells were observed between 26 and 36 hpf, specifically of cells arising from the primitive hematopoietic process, and the absence of appropriate tools to study the definitive wave, is that we focused in the effect of *csrnp1a* in primitive hematopoiesis. Finally, the expression domains described for both paralogs support the notion that the Csnrp family could have common roles in the biology of progenitor cells in the nervous system as well as in hematopoietic precursors. This suggestion is further sustained by our functional analysis.

### Csrnp1a Function during Early Zebrafish Development

At 30 hours post fertilization the phenotype of *csrnp1a* morphant embryos included a severe decrease in circulating blood cells, that is consistent with its expression in the ICM. This is reverted at 48 hpf, which is consistent with the observation that at 30 hpf the splicing morpholino ceases its function. This reversal could be explained because hematopoietic progenitors were not completely depleted or failed to differentiate and get arrested in an immature stage, and then when *csrnp1a* is processed again, the progenitors expands, differentiation resumes and the cells eventually go into circulation.

In addition, a generalized increase in cell death was observed in *csrnp1a* morphants, though this was considered nonspecific since co-injection with the p53 morpholino suppresses this effect. Importantly, when these embryos were screened at 32 hpf for reductions in blood cells in circulation, the proportion of embryos displaying the phenotype remained the same. This indicates that cell death is likely a consequence of morpholino toxicity and has no impact on the hematopoietic phenotype described.

In this study, we showed that Csrnp1a knockdown generates a developmental perturbation in the erythroid and myeloid primitive blood lineages, affecting *gata1* transcription and *spi1* message distribution respectively, however without effects on upstream genes like *klf4* or *tal1*.

### Csrnp1 and Csrnp1a Relationship

At early stages *csrnp1* and *csrnp1a* share expression domains suggesting a probable cross regulation or redundant functions. Csrnp1a knockdown does not impair *csrnp1* expression indicating that *csrnp1* is not a target of Csrnp1a, however, *csrnp1* morphants present the blood phenotype in 22,6% of the injected embryos. Since *csrnp1* is not expressed in the ICM at later stages, as is *csrnp1a*, this phenotype should derive from its early function. At 8 somites *csrnp1* is expressed covering the lateral plate mesoderm, where the hematopoietic progenitors arise [12]. In accordance to this, when *csrnp1a* and *csrnp1* morpholinos are co-injected the number of embryos presenting the blood phenotype increase up to 64,4%, pointing to a redundant role of both *csrnp* genes in hematopoiesis. This result and the fact that *csrnp1a* acts at 8 somites strength the notion that *csrnp1* is also exerting its hematopoietic function at these early stages.

### Erythropoietic Function of *csrnp1a*

In zebrafish, the first blood progenitors are specified in mesoderm during gastrulation. BMP4 first induces ventral-posterior mesoderm and subsequently directs a subpopulation of these cells towards the blood fate by activating *wnt3a*, *cdx* and *hox* genes [19]. These regulatory interactions induce the transcription factor, *tal1/scl*, which is central for the specification of hematopoietic precursors.

*tal1/scl* is expressed in hemangioblast, undifferentiated cells capable to produce blood and vascular lineages [16]. This protein form complexes with Lmo2, Gata2 and Gata1, controlling the expression of a variety of hematopoietic genes [20]. Downstream of these are discriminating factors essential for erythroid and myeloid development, as *gata1* and *spi1* respectively. *Krüppel-like, klf4*, is another important transcription factor that promotes *gata1* activation by direct binding to its promoter [3]. Our observations indicate that the observed inhibition of *gata1* in Csrnp1a morphants is not caused by deficiencies in *klf4* expression. Furthermore, the differential defects produced over *gata1* and *spi1* expression at the 8–12 somite stage, strongly suggests that at this time Csrnp1a could be part of a Tal1/Scl complex implicated in erythroid differentiation, specifically regulating *gata1* transcription.

It has been shown that *gata1* is also controlled by the transcription elongation factor DSIF. The *spt5/*Foggy mutant revealed that DSIF complex promotes *gata1* transcription [11]. It has been speculated that DSIF could recruit P-TEFb conferring functional specificity. P-TEFb also interacts with hematopoietic transcription factors such as Tif1-γ, LDB1, and Gata1, which are components of the Scl complex [11]. The observation that *Drosophila* Axud1 interacts in a double hybrid assay [10] and genetically with Spt5 (Glavic, unpublished result) reinforces the notion that Csrnp1a could be part of the Scl complexes involved in erythroid differentiation and *gata1* expression.

### Myelopoietic Function of Csrnp1a

The transcription factor *spi1* is a key factor in the myeloid linage. Our results show that Csrnp1a knockdown produces a distortion in its expression pattern, which does not seem to be solely due to transcriptional inhibition. The Scl complex in anterior lateral plate mesoderm regulates the myeloid specific factor *spi1*; here the specification of *spi1+* cells relies on the BMP pathway [21]. Another factor acting on *spi1* is retinoic acid. Embryos exposed to it show a decreased expression of *tal1*, *lmo*, *gata2, and eprs* in anterior hemangioblasts. Finally, and analogously to the erythroid lineage decisions, these factors drive the differentiation of anterior LPM cells to neutrophils and macrophages inducing the expression of markers like *mpo* or *lcp* [22].

The actual model of cell fate decisions between myeloid and erythroid lineages involves a cross negative regulation between *spi1* and *gata1* [22]. Recent data has added an extra layer to this regulation, the *tif1*-*γ* transcription factor, which according to the context can repress or activate *spi1* and *gata1*. At early stages *tif1-γ* morphants show a decrease in the expression of both genes [23].

Our results indicate that Csrnp1a influences, at different stages and independently, the development of erythroid and myeloid lineages without affecting the common progenitor reveled by *tal1* expression. A potential explanation to this arises considering developmental time: the fact that *tif1-γ* is capable to decrease both factors at early stages and that *csrnp1a* interacts with Spt5, a member of the DSIF complex [10], [Glavic unpublished results]. DSIF interacts with the positive (P-TEFb) and negative elongation factors (NELF) to exert its function, and on the other hand P-TEFb binds to *tif1-γ* and the Tal1/Scl complex to regulate erythroid development [24]. Although DSIF has not been implicated yet in early myeloid, the abovementioned data let us speculate that Csrnp1a, and possibly Tif1-γ, control over *spi1* arise from their interactions with Scl complex that work at discrete locations and stages of hematopoiesis recruiting P-TEFb, which finally acts through the DSIF complex.

As mentioned before, Csrnp1a did not affect *tal1* expression, and accordingly no alterations in endothelial markers or vessels morphology were detected. In addition, knocking down this gene did not disturb heart development, however we found a slight reduction in the heartbeat in morphant embryos. We consider this reduction too small to account for the very significant decrease in circulating blood cells. This is supported by the fact that blood cell flow density (number of cells in circulation moving through the visual field in a certain period of time) is almost null in morphant embryos, indicating that the problem is not based on blood flow but rather on the quantity of cells in circulation. Taken together all these data suggest that Csrnp1a has no influence in blood flow, and therefore the clear reduction in circulating blood cells probably arises as a specific role in primitive hematopoiesis.

In this paper we described new data regarding the Csrnp protein family in vertebrate development, specifically investigating zebrafish Csrnp1a. We have established a new role for this transcription factor family in primitive hematopoiesis. Further experiments are needed to precisely define the role of Csrnp1a in zebrafish development and hematopoiesis and its participation in the transcriptional network controlling it. Special attention requires the relation of Csrnp1a with Spt5 and the formation and activity of selective Scl complexes during erythroid and myeloid differentiation.

## Materials and Methods

### Ethics Statement

All animals subjected to experimentation were anesthetized and procedures complied with the guidelines of the Animal Ethics Committees of the Universidad Andres Bello and Universidad de Chile, which approved this study.

### Zebrafish Maintenance

Zebrafish were maintained and raised in our facility according to standard protocols [25]. The following fish strains were used in this study: Tab5 (wild type), *Tg(Bacmpx:GFP)^i114^* [26] and *Tg(fli1a:EGFP)^y1^* [27]. All embryos were collected by natural spawning, staged according to Kimmel *et al*. 1995 [28], and raised at 28,5°C in E3 medium (5 mM NaCl, 0.17 mM KCl, 0.33 mM CaCl2, 0.33 mM MgSO4, without methylene blue, equilibrated to pH 7.0) in Petri dishes, as described previously [25]. Embryonic and larval ages are expressed in hours post fertilization (hpf).

### Embryos Micro-injections and S-nitroso-N-acetyl-penicilamine (SNAP) Incubation

Injections were carried out on 1- to 2-cell stage embryos. To repress *csrnp1a* gene expression, two morpholinos were designed (Gene Tools), an ATG-targeting (Mocsrnp1a atg) 5′- ACTGACGAGCACAACTCAACAACAG -3′ to impair *csrnp1a* translation and a splicing morpholino (Mocsrnp1a spt) 5′-TATCATGAGTGACTTACTTGGCATG -3′ targeting the exon 2/intron 2 splice site. Mocsrnp1a atg was used at 2.5 ng per embryo and Mocsrnp1a spt at 5 ng per embryo. Both morpholinos were diluted in distilled water. To test the genetic redundancy between *csrnp1* and *csrnp1a* we used a csrnp1 atg morpholino at the concentrations previously described [12]. Also we used a control morpholino (5′-CAATCTGAGCATCTTACCTGAGGTG-3′) [29] injected at 2.5 and 5 ng. To check the effectiveness of Mocsrnp1a spt we observed the decrease of Csrnp1a mRNA through RT-PCR using cDNA made from splicing morphants and compared with control embryos. The *p53* morpholino (5′-GCGCCATTGCTTTGCAAGAAGAATTG -3′) was injected at the concentration described by Robu *et al*. 2007 [18].

Zebrafish embryos were incubated in 10 uM of SNAP between 10 somites and 23 hpf in E3 medium. Dimethyl sulfoxide (DMSO) carrier content was 0.1%.

### RT-PCR and qPCR

Total RNA was extracted from embryos at 0, 10, 19, 24, 30, 36, 48 hpf. 50 embryos of any of these stages were homogenized in TRIzol solution (Invitrogen) following manufacturer’s instructions and cDNA generated using Superscript II reverse transcriptase (Invitrogen) and oligo dT (Invitrogen) following also manufacturer’s instructions. PCR was conducted on resulting cDNA using the primers: Csrnp1aF: 5′-CTCTGATGAAGACAGTCCACAAAG-3′ and Csrnp1aR: 5′-TCAGAGTCCGAAGACAGACTGAG-3′ to generate a fragment of 1.2 kb. PCR conditions were 95°C 5 minutes, 30 cycles of 95°C 30 seconds for denaturation, 55°C 30 seconds for annealing and 72°C 1,5 minutes for extension, an extra extension at 72°C 15 minutes was included at the end of the procedure. Csrnp1 primers and RT-PCR was performed as previously described [12]. Real-time PCR was performed with the Mx3000P system (Stratagene) using SYBR Green. Samples contained 7,5 µL of Maxima SYBR Green qPCR Master Mix 2X with Rox (Fermentas), 0,5 µL of each primer (10 µM), 1 µL of template and 5,5 µL DEPC water. In negative controls cDNA was replaced by DEPC water. All PCR were done with 3 biological replicates. The Real-time conditions were 95°C for 10 min and 40 cycles of 95°C 30 seconds, 56°C 30 seconds and 72°C 30 seconds using the primers: *β-actin*F 5′-ATCTTCATCAGGTAGTCTGTCAGGT-3′, *β-actin*R 5′-AAGGCTTCCAGTTTTTCCTCCC-3′, *csrnp1a*F 5′-CTCTGATGAAGACAGTCCACAAAG-3′ and *csrnp1a*R 5′-GTAACCGAACCAAACCGAAC-3′.

### Whole Mount *in situ* Hybridization and Immunohistochemistry

ISH experiments were performed as described in Thisse et al. 2008 [30]. The RNA-probes were sensitized according to standard protocols. *csrnp1a* probe was synthesized using the AGENCOURT_22438433 EST (OpenBiosystems) as template. The following genes were kindly provided as cDNA clones and used as templates for making RNA probes: *ntl* [31], *gsc* [32], *gata5* [33], *tal1/scl* [6], *klf4* [11], *spi1/pu.1* [6], *gata1* [34], *hbae1* [35], *lcp1* [36], *amhc* [37], *vmhc* [37] and *myoD* [38]. For immunohistochemistry, we used rabbit Antiphospho-Histone H3 (H3-P, Upstate 07–424). Immunolabeling was carried out essentially as described in Sarrazin et al. 2006 [38]. To define the region for H3-P-positive cell counts, immunohistochemistry against H3-P was performed in embryos previously labeled by *in situ* hybridization with *spi1/pu.1* or *scl* probes. H3-P signal was only counted inside the *in situ* area.

### O-dianisidine and DAB Stains

O-dianisidine stain was performed as previously described [39]. The DAB stain solution was prepared as follow; 0.07 mM of 3,3′diaminobenzidine dissolved in 300 ul DMSO, 533 ul ethanol, 450 ul PBS and 50 ul H_2_O_2_. Embryos were fixed 2 hours in 4% PFA/PBS, washed 5 minutes with water, then 3 washes of 5 minutes in PBST, one more in PBS and finally incubated in the DAB stain solution. Embryos were observed after 3 hours of incubation.

### Acridine Orange Staining

Embryos were stained according to Williams and Webb, 2000 [40]. Briefly, embryos were incubated for 20 min in 5 ug/ml acridine orange (Sigma) in E3 medium, washed five times for 5 min in E3 medium and observed under fluorescence microscopy.

## Supporting Information

Figure S1 ***csrnp1a*** **gene scheme, morpholino target sites, primers used and RT-PCR of** ***csrnp1a*** **.** The complete *csrnp1a* genome sequence comprises 14,724 bp, which contains five exons (blue boxes) and 6 introns (red boxes). In the diagram morpholino hybridization sites are represented with black lines and in green lines represent the primers hybridization sites designed for RT-PCR. (B) RT-PCR of 24 hpf control embryos (C) and Splicing morphants (MoSpt) injected with 2,5 and 5 ng per embryo, showing the dose dependent decrease of *csrnp1a* mRNA. This decrease was reverted at 30 hpf, indicating that the spt morpholino is no longer efficient at this time.
(TIF)

Figure S2 **Early mesoderm specification is not altered in** ***csrnp1a*** **knockdown condition.** Expression of the mesodermal markers *goosecoid* (*gsc*) (A, D), *notail* (*ntl*) (B, E) and *gata5* (C, F) detected by ISH at three different stages of gastrulation. No differences were detected between morphant and control embryos (100%, n = 37, 49 and 46 embryos respectively).
(TIF)

Figure S3 ***csrnp1a*** **knockdown does not affect blood flow.** Morphant embryos were incubated in the vasodilator SNAP, and its ability to rescue the reduction of blood cells was analyzed (A, B, C). *csrnp1a* knockdown phenotype was not rescue by SNAP treatment. (A) Control 100% n = 53; (B) Mo csrnp1a atg 36,8% n = 21/57 of morphant phenotype; (C) Mocsrnp1a atg incubated with SNAP 33,9% n = 21/62 of morphant phenotype. *In situ* hybridizations against ventricular (*vmhc*) (D, E) and auricular (*amhc*) (F, G) markers show that injected embryos have normal heart development (100%; n = 43; n = 45 respectively). All are lateral views, anterior to the left. (H) Chart depicting the number of heartbeat in 10 seconds in control and *csrnp1a* morphant embryos.
(TIF)

Figure S4 ***csrnp1a*** **morphants exhibit an increase in cell death due to morpholino toxicity which is not responsible for the reduction in blood cells in circulation.** We analyzed cell death by acridine orange in embryos injected with control morpholino (A), *csrnp1a* morpholino (C), *p53* morpholino (E), or a mixture of *csrnp1a* and *p53* morpholinos (G). A clear increased in cell death is detected in *csrnp1a* morphant embryos (86,7%; n = 58/67), which is reversed by *p53* co-injection (16,7%; n = 16/96). The cell blood phenotype was screened at 33 hpf using O-Dianisidine stain in (B) control, (D) *csrnp1a*, (F) *p53* morphant embryos and in embryos co-injected with both morpholinos (H). The *csrnp1a, p53* co-injected embryos (H; 39,6% n = 21/53) have the same penetrance of the blood phenotype as *csrnp1a* morphants (F; 35,7% n = 15/42) (red arrows). It worth mention that the slight head reduction exhibited by *csrnp1a* morphant embryos was also detected in co-injected embryos (black arrows). All are lateral views, anterior to the left.
(TIF)
