# Supplementary figures and images for: csrnp1a Is Necessary for the Development of Primitive Hematopoiesis Progenitors in Zebrafish
Source: PLoS One. 2013 Jan 9;8(1):e53858. doi: 10.1371/journal.pone.0053858 (PMC3541188; doi:10.1371/journal.pone.0053858)

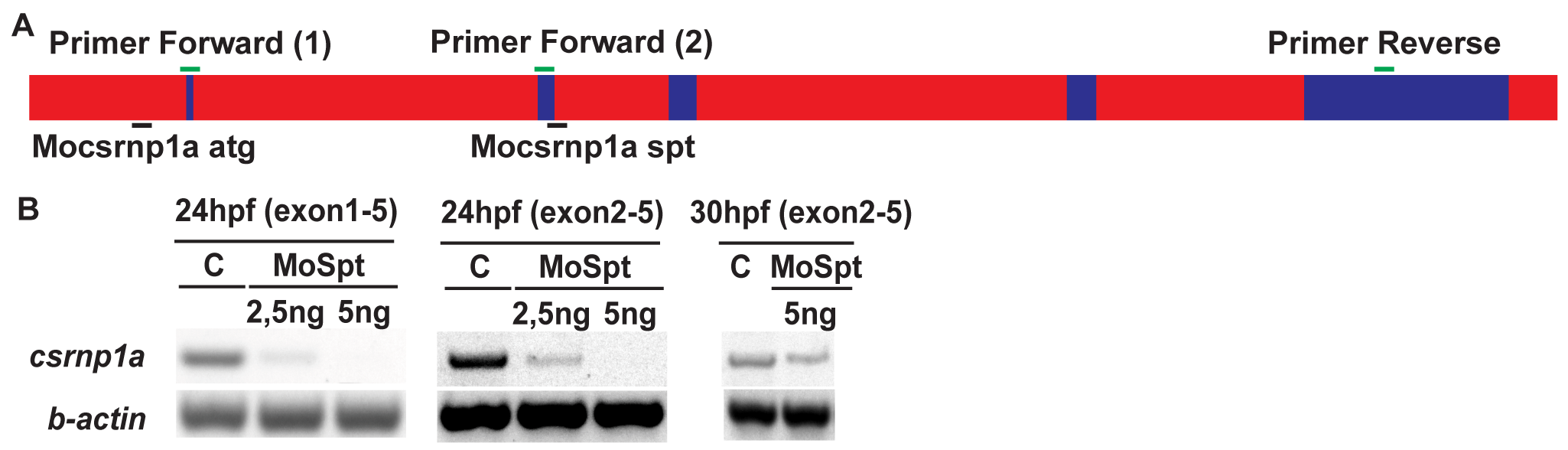

Supplement: Figure S1 — csrnp1a gene scheme, morpholino target sites, primers used and RT-PCR of csrnp1a . The complete csrnp1a genome sequence comprises 14,724 bp, which contains five exons (blue boxes) and 6 introns (red boxes). In the diagram morpholino hybridization sites are represented with black lines and in green lines represent the primers hybridization sites designed for RT-PCR. (B) RT-PCR of 24 hpf control embryos (C) and Splicing morphants (MoSpt) injected with 2,5 and 5 ng per embryo, showing the dose dependent decrease of csrnp1a mRNA. This decrease was reverted at 30 hpf, indicating that the spt morpholino is no longer efficient at this time. (TIF) [file pone.0053858.s001.tif]

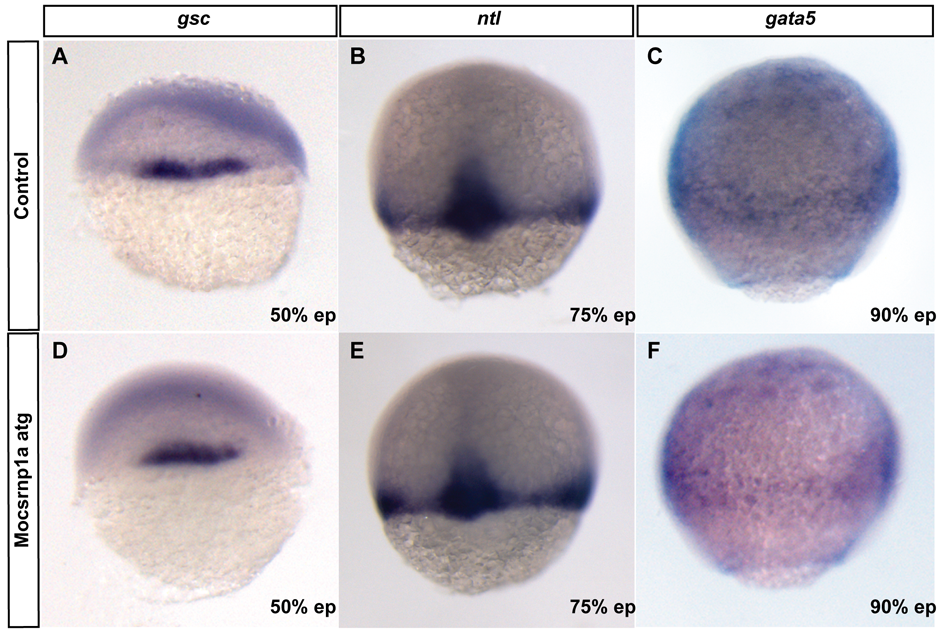

Supplement: Figure S2 — Early mesoderm specification is not altered in csrnp1a knockdown condition. Expression of the mesodermal markers goosecoid (gsc) (A, D), notail (ntl) (B, E) and gata5 (C, F) detected by ISH at three different stages of gastrulation. No differences were detected between morphant and control embryos (100%, n = 37, 49 and 46 embryos respectively). (TIF) [file pone.0053858.s002.tif]

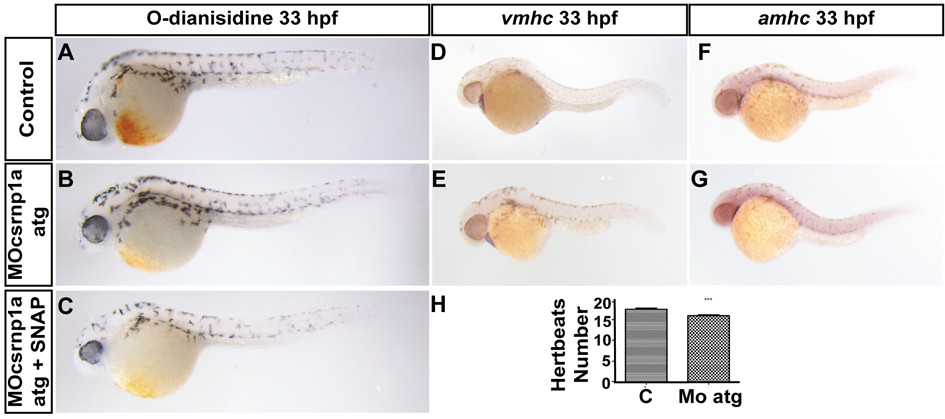

Supplement: Figure S3 — csrnp1a knockdown does not affect blood flow. Morphant embryos were incubated in the vasodilator SNAP, and its ability to rescue the reduction of blood cells was analyzed (A, B, C). csrnp1a knockdown phenotype was not rescue by SNAP treatment. (A) Control 100% n = 53; (B) Mo csrnp1a atg 36,8% n = 21/57 of morphant phenotype; (C) Mocsrnp1a atg incubated with SNAP 33,9% n = 21/62 of morphant phenotype. In situ hybridizations against ventricular (vmhc) (D, E) and auricular (amhc) (F, G) markers show that injected embryos have normal heart development (100%; n = 43; n = 45 respectively). All are lateral views, anterior to the left. (H) Chart depicting the number of heartbeat in 10 seconds in control and csrnp1a morphant embryos. (TIF) [file pone.0053858.s003.tif]

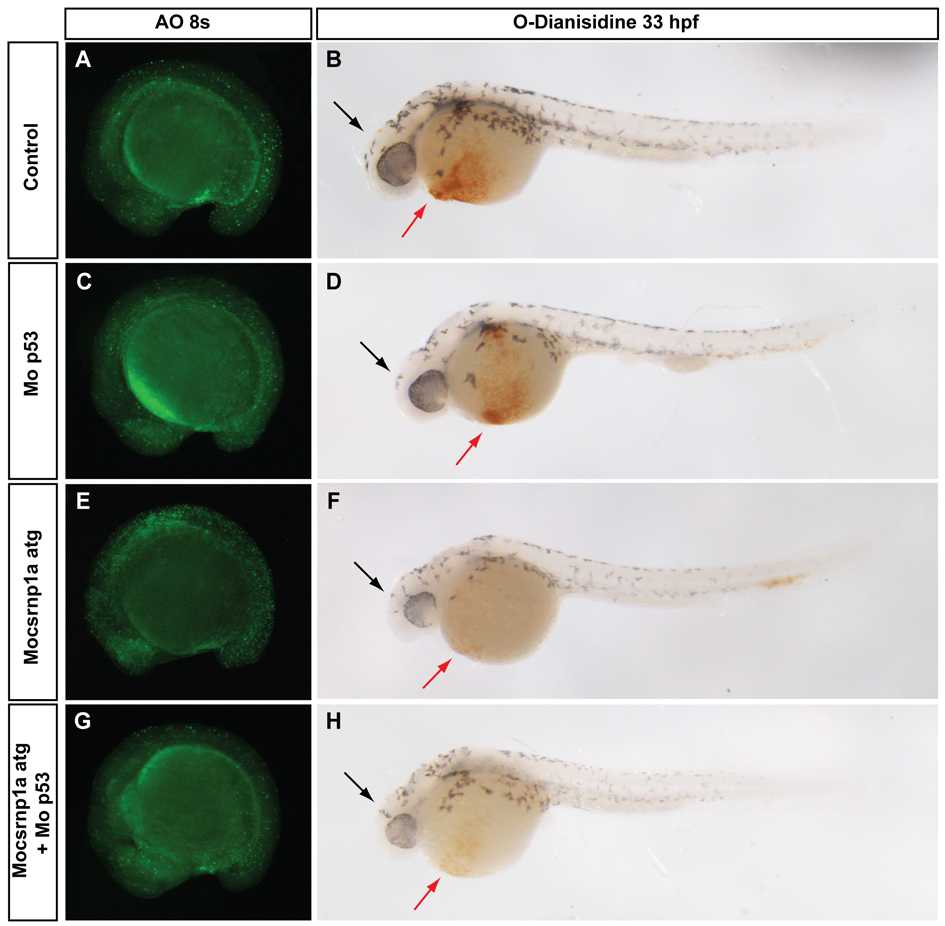

Supplement: Figure S4 — csrnp1a morphants exhibit an increase in cell death due to morpholino toxicity which is not responsible for the reduction in blood cells in circulation. We analyzed cell death by acridine orange in embryos injected with control morpholino (A), csrnp1a morpholino (C), p53 morpholino (E), or a mixture of csrnp1a and p53 morpholinos (G). A clear increased in cell death is detected in csrnp1a morphant embryos (86,7%; n = 58/67), which is reversed by p53 co-injection (16,7%; n = 16/96). The cell blood phenotype was screened at 33 hpf using O-Dianisidine stain in (B) control, (D) csrnp1a, (F) p53 morphant embryos and in embryos co-injected with both morpholinos (H). The csrnp1a, p53 co-injected embryos (H; 39,6% n = 21/53) have the same penetrance of the blood phenotype as csrnp1a morphants (F; 35,7% n = 15/42) (red arrows). It worth mention that the slight head reduction exhibited by csrnp1a morphant embryos was also detected in co-injected embryos (black arrows). All are lateral views, anterior to the left. (TIF) [file pone.0053858.s004.tif]
